# Persistent Inflammation in Pulmonary Granuloma 48 Years after Talcage Pleurodesis, Detected by FDG-PET/CT

**DOI:** 10.1155/2012/686153

**Published:** 2012-11-06

**Authors:** J. C. Fanggiday, R. W. Rouse, S. M. Collard, M. J. de Haas, J. M. H. de Klerk

**Affiliations:** ^1^Department of Nuclear Medicine, Meander Medical Center, Utrechtseweg 160, 3818 ES Amersfoort, The Netherlands; ^2^Department of Pathology, Meander Medical Center, Utrechtseweg 160, 3818 ES Amersfoort, The Netherlands; ^3^Department of Pulmonology, Meander Medical Center, Utrechtseweg 160, 3818 ES Amersfoort, The Netherlands

## Abstract

In patients with suspicion of lung malignancy, FDG PET/CT is frequently used as a diagnostic and staging imaging modality. However, false positive findings are not uncommon. We demonstrate a case with FDG-avid pulmonary nodules, mimicking lung cancer. After histopathological examination they appeared to be the result of persistent inflamed tissue, due to talcage pleurodesis, which occurred 48 years ago. We concluded that, nearly five decades after talcage pleurodesis, there can still be an ongoing inflammation reaction in the pleurae, which can be detected by FDG PET/CT.

## 1. Case Report

A 68-year-old male, nonsmoker, visited the outpatient clinic of the urologist. He is known with a carcinoma of the prostate, cT1c Gleason score 6, tumour-load less than 10%, right sided. Abdominal CT revealed no signs of lymphogenous or distant metastasis. Transrectal ultrasonography and clinical evaluation showed no signs of local invasion, so a strategy of active surveillance has been proposed. Further medical history revealed a spontaneous left-sided pneumothorax, treated with talcage pleurodesis at the age of 20. There were no asbestos exposure, no known exposure of tuberculosis.

After a period of cough, chest radiography has been performed, which showed nodular structures, subpleural in the left upper lobe and just lateral of the thoracic aorta. The patient was sent by the general practitioner to the pulmonologist and due to his medical history and the result of the chest radiography an FDG-PET/CT has been made.

**Figure 1:**
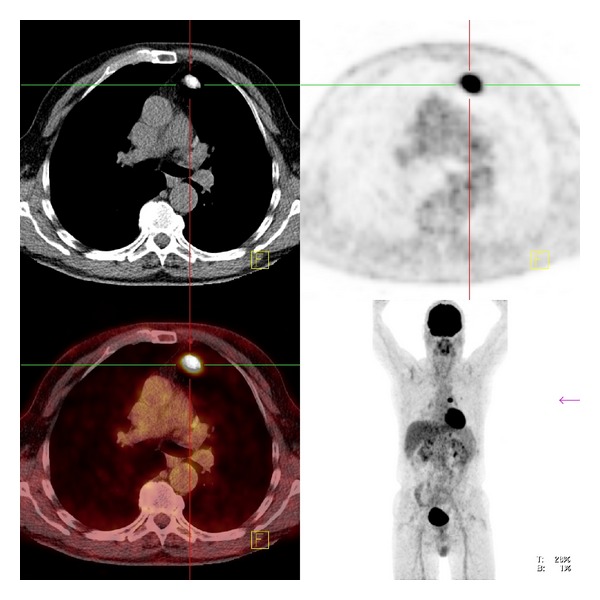
Pulmonary nodule with high FDG uptake ventrally in the left upper lobe.

**Figure 2:**
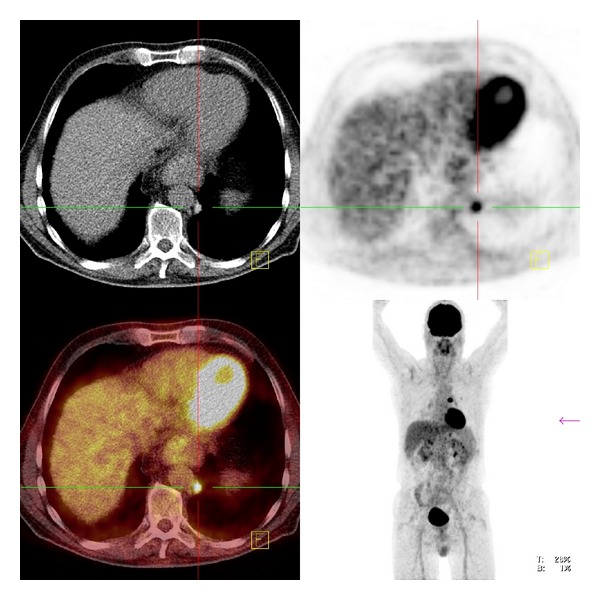
FDG-avid paraaortic nodal structure.

Computed tomography showed indeed a round, dense structure ventral in the left upper lobe, with an HU value of 168 (Figure 1). The paraaortic nodule revealed an HU value of 132 (Figure 2). All these nodules showed a high FDG uptake, with respectively maximum standard uptake values (SUVmax) of 16.4 and 7.7. At some places the pleurae appeared to be thickened and hyperdense at CT, with also high FDG uptake (SUV max 4.7). No abnormalities elsewhere were observed, especially no hilar or mediastinal lymphadenopathy. No signs of emphysema.

**Figure 3:**
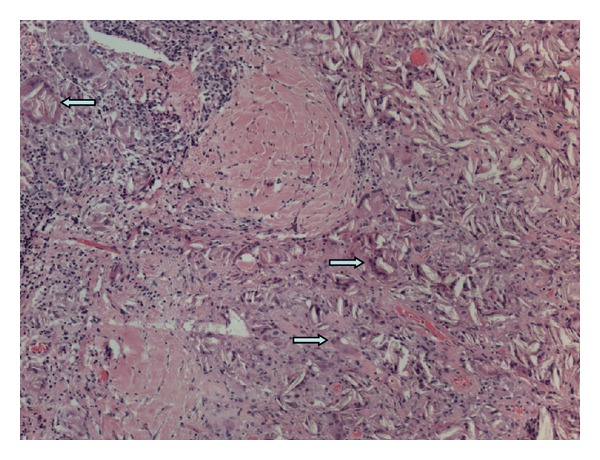
A representative low-power (H&E, ×200) photograph showing crystals surrounded by multinucleated foreign-body giant cells (arrows).

**Figure 4:**
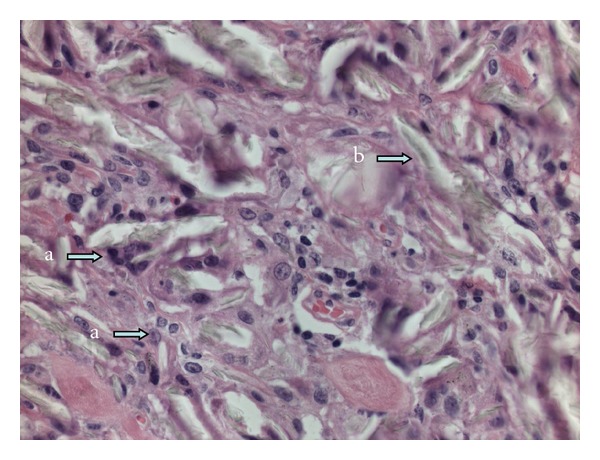
High-power view (H&E, ×400) of Figure 3, showing multinucleated foreign-body giant cells (a) and crystals (b).

**Figure 5:**
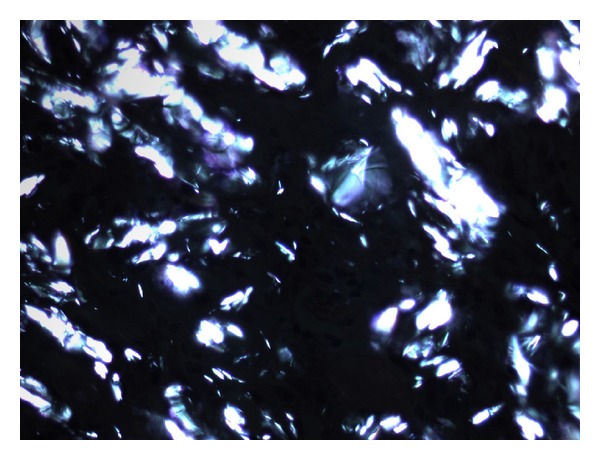
High-power view (H&E, ×400) of polarized light microscopy of Figure 3, showing the birefringent characteristics of the crystals.

Patient underwent a video assisted thoracoscopy. The upper lobe appeared to be stitched with the mediastinal wall. The ventral nodule was localized and excised. Pathology showed a nodule mainly consisting of lung and pleural tissue, and furthermore, multinucleated foreign-body giant cells and histiocytes reaction with many birefringent crystals. No signs of malignancy (Figures 3, 4, and 5) were observed. This is in accordance with a foreign-body reaction with ongoing inflammation.

The paraaortic nodule has not been excided. However, follow-up FDG-PET/CT scan after half a year showed no changes in CT and FDG-PET characteristics of this nodule.

## 2. Discussion

Since the introduction of talc pleurodesis by Bethune in 1935, this procedure is used to manage recurrent malignant and nonmalignant pleural effusions and recurrent or persistent pneumothoraces [1]. Talc is hydrated magnesium silicate and causes an intrapleural inflammatory response. Adhesion molecules, cytokines IL-8, VEGF, and TGF-beta are formed. This response is thought to cause adhesions [2, 3]. In 1969, Jones already described the effect of talcage pleurodesis, where as early as three weeks after the procedure talc granulomata were present [4].

Talc pleurodesis has proven its role in treatment of pneumothoraces [5]. There are no or minimal long-term side effects in these patients [6, 7]. Computed tomography appearances of talc pleurodesis are well described [8, 9]. Results of talc pleurodesis can be seen at CT examination as lesions with high attenuation, in or nearby the pleurae. Murray also hypothesized the FDG uptake in these lesions with granulomatous inflammation. In his series, FDG uptake was seen up to 10 months after pleurodesis [10]. Since that report, several authors described the FDG-PET appearances of talc pleurodesis. During followup, standard uptake values appeared to persist or increase further [11]. Several case reports described an increased FDG uptake, even years after initial talc pleurodesis. Peek et al. for example, described increased FDG uptake 10 and 11 years, respectively, after talc pleurodesis. Follow up was done without histological proof, but by CT, which showed no signs of malignancy after 16 months [12]. 

In our case both CT and FDG-PET findings fitted well with the characteristics described above. However, such a long interval after talcage biopsy with histologically proven diagnosis, to the best of our knowledge, has not been described before.

## 3. Conclusion

Pulmonary FDG-avid nodules due to talcage pleurodesis can occur even after almost fifty years after the procedure.
